# Early experiences with usage of long‐acting injectable cabotegravir among adults in rural Ugandan and Kenyan communities: qualitative research from the SEARCH “Dynamic Choice HIV Prevention” intervention trials

**DOI:** 10.1002/jia2.70059

**Published:** 2025-11-24

**Authors:** Carol S. Camlin, Anjeline Onyango, Jason Johnson‐Peretz, Cecilia Akatukwasa, Titus O. Arunga, Lawrence Owino, Frederick Atwine, Ambrose Byamukama, Andrew Mutabazi, Laura B. Balzer, Maggie Czarnogorski, Elijah Kakande, Helen Sunday, Gabriel Chamie, James Ayieko, Maya Petersen, Nicole Sutter, Moses R. Kamya, Diane V. Havlir, Jane Kabami

**Affiliations:** ^1^ Division of Prevention Science Department of Medicine, University of California, San Francisco (UCSF) San Francisco California USA; ^2^ Obstetrics, Gynecology & Reproductive Sciences University of California, San Francisco (UCSF) San Francisco California USA; ^3^ Kenya Medical Research Institute (KEMRI) Kisumu Kenya; ^4^ Infectious Diseases Research Collaboration Kampala Uganda; ^5^ Berkeley, Biostatistics, Epidemiology, and Computational Precision Health University of California Berkeley California USA; ^6^ ViiV Healthcare Washington DC USA; ^7^ Division of HIV, Infectious Diseases & Global Medicine Department of Medicine, University of California, San Francisco (UCSF) San Francisco California USA; ^8^ Department of Medicine Makerere University College of Health Sciences Kampala Uganda

**Keywords:** HIV prevention, HIV pre‐exposure prophylaxis, PrEP, long‐acting cabotegravir, CAB‐LA, Kenya, Uganda

## Abstract

**Introduction:**

Despite oral HIV pre‐exposure prophylaxis (PrEP) effectiveness, uptake and adherence remains a challenge. Newer HIV prevention technologies, including long‐acting injectable cabotegravir (CAB‐LA), are promising for addressing known barriers to oral PrEP uptake and adherence, yet research remains limited on experiences with CAB‐LA among at‐risk adults in community settings. This descriptive qualitative study explored experiences with CAB‐LA among adults participating in the SEARCH Dynamic Choice HIV Prevention (DCP) trial in rural Kenya and Uganda, which evaluated HIV prevention uptake through a structured, person‐centred DCP model.

**Methods:**

We conducted in‐depth semi‐structured interviews in July−October 2023 with a purposively selected sample of 47 DCP trial participants who initiated CAB‐LA and had at least two injections. Interviews explored participants’ reasons for choosing CAB‐LA and their experiences with the method. We also included 10 participants who subsequently discontinued CAB‐LA or switched to another method. Data were analysed using inductive coding, memoing and framework analysis.

**Results:**

The 47 participants ranged from 20 to 59 years of age; 13 were men and 34 were women. Participants were enthusiastic about CAB‐LA. They perceived it as novel, efficacious and advantageous relative to oral daily PrEP, which had been hindered by stigma, interruptions due to work, family visits and travel, side effects, and pill attributes (size and smell). Two major advantages of CAB‐LA over PrEP were improved protection from HIV stigma and from HIV acquisition due to easier adherence. Participants felt CAB‐LA was clearly distinguishable from antiretroviral therapy and would not mark them (mistakenly) as living with HIV; among women, clandestine use to guard against stigma from family members was more achievable compared to oral PrEP. Appointments for injections were rare enough (monthly, then bimonthly) that they could be kept, especially with reminders from providers, although for some, unpredictable work and travel schedules hindered their uptake of CAB‐LA. Participants cited injection‐site pain as the main drawback.

**Conclusions:**

CAB‐LA overcame several known barriers to HIV prevention uptake and adherence for women and men. In contexts of continued HIV‐related stigma, CAB‐LA met some participants’ preferences for a product that permitted prevention to be visibly distinguishable from treatment, enabling HIV prevention uptake to feel safer. Moreover, adherence was more easily achieved with CAB‐LA compared to PrEP, boosting confidence in prevention efficacy.

## 1 INTRODUCTION

Despite the efficacy of oral HIV pre‐exposure prophylaxis (PrEP) for HIV prevention, PrEP uptake, adherence and persistence have remained suboptimal. Barriers to adherence include daily pill burden, fear of stigma, forgetfulness and fear of disclosure to partners, especially among women [1, 2]. Long‐acting cabotegravir (CAB‐LA) was approved by the US FDA in 2021 and recommended by the WHO in 2022 to widen the range of effective HIV prevention options available to those at risk of HIV acquisition [3].

CAB‐LA has the potential to address challenges to uptake and adherence to oral PrEP. It addresses the burden of daily pill‐taking associated with oral PrEP and can more easily be used clandestinely [1, 2, 3, 4]. Thus, it offers substantial benefits to those seeking to avoid HIV and PrEP‐related stigma and to those who wish to conceal their usage from sexual partners (e.g. to avoid relationship conflict or discussions around fidelity). CAB‐LA was approved in Kenya in 2024, and the Kenyan Ministry of Health (MoH) anticipates the medication to be in the country by the end of 2025; in Uganda, the MoH approved CAB‐LA as an HIV prevention option in 2024 [5], but it is not yet widely available in health facilities.

Rollout of CAB‐LA has been met with expressions of concern that undetected HIV at initiation or HIV acquisition during the pharmacokinetic tail after initiating and discontinuing CAB‐LA could result in resistance to dolutegravir and compromise the effectiveness of dolutegravir‐based first‐line treatment regimens [1, 6, 7]. CAB‐LA exposure could result in resistance if administered to persons who have newly acquired HIV when preliminary tests are not sensitive enough for early HIV diagnosis. Critics cite higher costs and potential INSTI (integrase nucleoside strand transfer inhibitor) resistance concerns, though observations to date have not confirmed these risks [1, 3]. Studies show that resistant breakthrough infections can occur but have been rare, and modelling studies show that the long‐term benefits of CAB LA far outweigh its risks [3, 7]. Studies have also suggested that CAB‐LA can be made sustainable if the price is lowered, if it is made widely available or if its delivery is moved as close to the clients as possible [6, 7, 8]. Extension of the period between injections also has the potential to improve sustainability, as promised by the twice‐per‐year injectable Lenacapavir [6, 7] and other products in development like cabotegravir ultra long‐acting (CAB‐ULA), which can be dosed at intervals of at least 4 months [9].

CAB‐LA was highly acceptable and preferred over oral PrEP, especially by HPTN 077 clinical trial cohort participants outside the United States [2]; HPTN 084, among African women, similarly found that participants preferred injections to pills [10]. Limited research exists to date on the preferences, experiences and barriers to injectable PrEP use among men and women offered CAB‐LA in sub‐Saharan Africa [2, 3, 10, 11, 12]. We, therefore, conducted a qualitative analysis to assess early experiences with CAB‐LA delivered at government clinics during a study of dynamic choice HIV prevention in rural Kenya and Uganda, where individuals had a choice of HIV prevention methods and could switch methods when they chose to do so. Understanding preferences and decision‐making pathways will help inform future rollout and scale‐up of CAB‐LA and other injectable HIV prevention options in these communities and similar settings.

## 2 METHODS

### 2.1 Study context

This qualitative study of people who chose CAB‐LA as a prevention method took place within the Dynamic Choice HIV Prevention (DCP) intervention of the SEARCH‐SAPPHIRE Study (NCT05549726), testing innovative strategies for HIV prevention to reach populations at risk of HIV acquisition with scalable interventions [13, 14, 15, 16]. The DCP intervention was implemented by clinical officers and nurses at antenatal clinics (ANCs), outpatient departments (OPDs) and settings served by community health workers (CHWs) across eight communities in rural southwest Uganda and western Kenya [13]. The DCP intervention offered participants a choice of HIV prevention options, including HIV self‐test kits, condoms, PrEP and post‐exposure prophylaxis (PEP). In 2023, following national approval of CAB‐LA in Kenya and Uganda, the study began to roll out injectable CAB‐LA for these participants as part of the DCP package [14]. Clients had 24/7 (hours/days) access to clinicians via mobile phones and received personalized plans to address challenges to uptake and adherence, and were offered integrated reproductive health services.

### 2.2 Ethical approval and informed consent

The study was approved by the Kenya Medical Research Institute (KEMRI 4173), the Makerere University School of Medicine Research and Ethics Committee (REC‐REF 2020‐029), and the University of California San Francisco (UCSF) Committee on Human Research (20‐32144). All study participants provided written informed consent to be interviewed, prior to data collection.

### 2.3 Study sample

We purposively enrolled participants from the DCP trials as the intervention was implemented using categories of gender, community (balanced across Kenya and Uganda) and DCP trial recruitment site (OPD, ANC/PNC, CHW as noted in “Study Context”), ensuring also that we included approximately 10 participants who had opted to discontinue CAB‐LA after the first or second injection. Reasons for discontinuation ranged from travel out of the area to pregnancy, and participants had the option to switch to other methods [14]. We capped the number of discontinuers at 10 for sampling size reasons; however, the proportion of discontinuers in this qualitative study is about equal to that of the main study (21% and 24%). The qualitative study began recruitment of the adults who had completed their week 24 visit (i.e. at least two injections) in the DCP trials and continued until approximate balance across purposive sampling categories and adequate data saturation was obtained; the final number interviewed was *n* = 47. Enrolment began in July and continued through September 2023.

### 2.4 Data collection

A gender‐balanced team of trained qualitative researchers (two women, three men) who were native speakers of participants’ local languages (DhoLuo, Runyankole, Swahili and English) conducted in‐depth semi‐structured interviews with participants between July and October 2023. Interviews were conducted in private locations convenient for participants to ensure confidentiality. The interview guide explored experiences with DCP counselling, product preferences and reasons for those preferences, and factors in the decision to start CAB‐LA, including perceived HIV risk, partner/family support, stigma/disclosure, prior experiences with oral PrEP, expectations and experiences with CAB‐LA, product and quality of care satisfaction, barriers/facilitators to persistence and recommendations to improve CAB‐LA delivery. The guide is included as Supplementary Material.

### 2.5 Data analysis

The interviewers (CA, FA, TOA, AO, LO) transcribed and translated audio recordings. Data were analysed in phases using both thematic and framework analysis approaches. A preliminary in‐depth analysis of the initial 16 transcripts was undertaken by CSC and JJ‐P using inductive analysis and memoing, with full team review. The qualitative team (LO, AO, CA, FA, TOA, JJ‐P, CSC) then developed a codebook from *a priori* concepts based on the interview guides; inductive codes were added following Charmaz's two‐stage method [17], and integrated into a final codebook [18]. Six team members applied codes to transcripts (LO, AO, CA, FA, TOA, JJ‐P) using Dedoose software [19]. Questions during the coding process were discussed by the full team at fortnightly meetings until consensus was reached. After transcripts were coded, groupings of coded excerpts were reviewed and discussed by the full team in an in‐person meeting in Kenya in June 2024 (led by CSC), including two new team members who had not participated in interviewing or coding (AM, AB). The team then used a framework analysis approach [20] to organize coded data, refine emergent themes and summarize the findings presented below.

## 3 RESULTS

*“Let those who brought this HIV prevention option of injectable PrEP continue helping women. Women are really suffering; someone is faithful to her husband, yet he will end up infecting the wife with the virus. Mostly men are the route of HIV infection in a family or among married couples. That is my cry.” – 30 y.o. female, Kenya*

Table 1 shows characteristics of the purposive sample of clients interviewed in this study. The sample was balanced by country with heterogeneity in age, but was predominantly female. Most participants were married or co‐habiting with a partner (72%), and engaged in subsistence farming as a primary livelihood (46%).

**Table 1:** Characteristics of CAB‐LA interview participants

	Overall
	*N*=47
**Recruitment setting** ^a^	
‐Antenatal clinic	17 (37%)
‐Outpatient department	16 (35%)
‐Community	13 (28%)
**Country**	
‐Kenyan	26 (55%)
‐Ugandan	21 (45%)
**Sex**	
‐Female	34 (72%)
‐Male	13 (28%)
**Age**	
‐Age 15−24 years	16 (34%)
‐Age 25 years	31 (66%)
**Marital status** ^a^	
‐Single (unmarried)	9 (20%)
‐Married/cohabitating	33 (72%)
‐Divorced/separated/widowed	4 (9%)
**Occupation** ^a^	
‐Farmer	21 (46%)
‐Shopkeeper/market vendor	5 (11%)
‐Student	1 (2%)
‐Manual labour/construction	2 (4%)
‐Transportation	2 (4%)
‐Bar/hotel/restaurant	1 (2%)
‐Fishing/fishmonger	1 (2%)
**Pregnant** (female only)	2 (6%)
**Circumcised** (male only)^a^	7 (58%)
**Alcohol use** ^a^	15 (33%)
**Highly mobile** ^a^	7 (15%)

*Note*: Alcohol use defined as having one or more drinks per week. Highly mobile defined as having consecutively been away for 2 weeks at least twice in the last 12 months.

^a^ Missing data for one participant.

**Figure 1:**
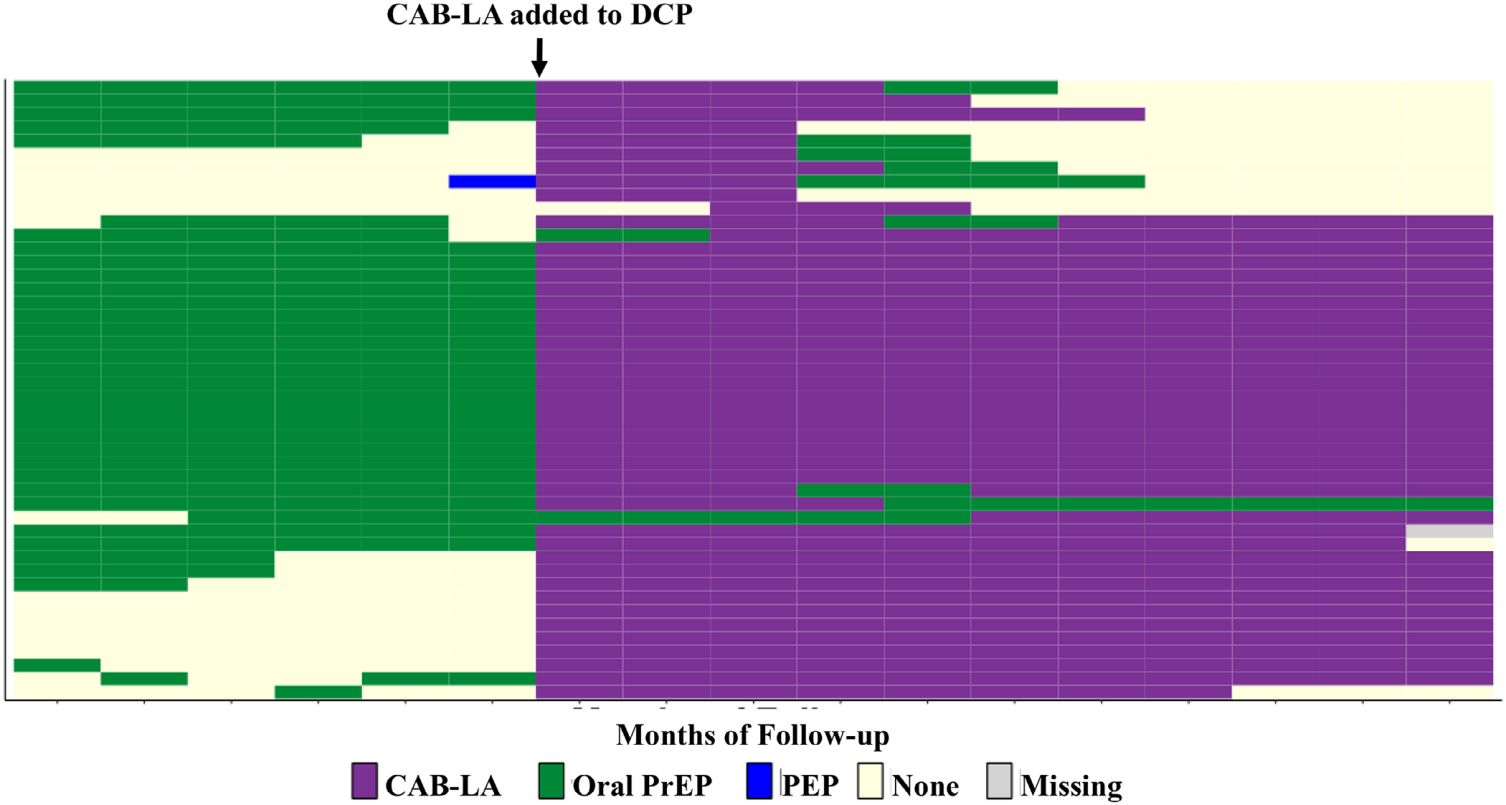
HIV prevention method usage over 18 months, qualitative study participants.

Figure 1 shows prevention product usage (by type) among qualitative study participants over the 6 months prior to CAB‐LA usage and 12 months following the introduction of CAB‐LA. Of those who took up CAB‐LA, 43% had not previously been on oral PrEP or PEP, while 57% switched from oral HIV prevention methods; 28% used two methods during the 48 weeks follow‐up period [14].

In the results below, we present clients’ motivations for taking up CAB‐LA, its advantages and challenges, and reasons for discontinuation when applicable.

### 3.1 Motivations

Several reasons motivated participants to start CAB‐LA, and many found they liked it for the reasons they had expected. Uptake appeared driven by peer influence, efficacy beliefs, anticipated privacy, a more manageable regimen due to its lasting nature and feeling protected in case of unplanned (or unwanted) sex.

#### 3.1.1 Novelty and peer influence

Participants cited the newness of the method as making it an attractive option, and were motivated to start CAB‐LA because many other people they knew were also initiating. One woman described initially telling her provider she was afraid of injections, then changing her mind:

…They told me, “If you are afraid of getting the injection, we can still give you the pills.” I told them, “No, why should I be the only one taking pills, [when] there is a new method? I will just use what everyone else is using. I will get used to it over time.” (39 y.o. female, Kenya)

#### 3.1.2 Efficacy beliefs

Participants felt confident that CAB‐LA, like oral PrEP, was effective and made them feel protected. For others, proof of efficacy came from their HIV‐negative test results: “I have never received any negative results [i.e., an HIV‐reactive test]. That is why I believe that the drug works.” (26 y.o. male, Uganda). Provider counselling and trust in doctors’ integrity also influenced participant choice. As one participant explained, “the provider told us that those people who had used the injection before in other places had been tested, and they were not infected with HIV. That is how it got introduced for us here in Uganda to also use. That gave me the confidence to use the method.” (26 y.o. female, Uganda). Another commented, “I know that doctors cannot approve of something bad.” (45 y.o. male, Kenya).

#### 3.1.3 Privacy

Another commonly cited motivation for starting CAB‐LA uptake was how it permitted participants to conceal their use of an HIV prevention method. Participants chose CAB‐LA because they had struggled to hide oral pills to avoid marital conflicts. PrEP had brought distrust between partners:

There are times when my husband chases me away from home for almost two days, and usually, I go and stay with my brother when I have not carried my medicine. I told the provider how I wished God would help us and [if] the injection was introduced, I would be happy. The provider told me that God is going to do what I am praying for. Sometimes, I would leave home to go and dig, and my husband would check everywhere looking for the medicine […] So I felt my heart settled with the injection. (44 y.o. female, Uganda)

While women more frequently than men discussed hiding PrEP pills, both male and female participants valued the ability to use CAB‐LA clandestinely to avoid potential conflicts with partners:

With the tablets, the health workers would come at home to teach us about it and I thought this [oral PrEP] was not appropriate for me, because I wanted to keep it between ourselves [myself and the provider] and sometimes it does not go well with my wife. So, the health worker agreed to meet me privately. (44 y.o. male, Uganda)

The desire for confidentiality was also tied to a desire to avoid the stigma resulting from other people confusing PrEP pills with HIV antiretroviral therapy (ART), and mistakenly concluding the person taking HIV prevention was living with HIV:

When you are among many people, it is difficult to take your pills, at times someone may check your bag and they land on the pills. It is difficult to explain to such people because the moment they see the pill bottle, they immediately conclude that I have HIV. I had to be careful all the time to prevent people from touching my bag and start to spread rumors. But with the injections, you are safe. (36 y.o. female, Uganda)

#### 3.1.4 Long‐acting

Participants also chose to start CAB‐LA specifically because of its long‐acting features and less taxing regimen. For some, CAB‐LA was easier to use because it did not interfere with work and travel; one did not need to remember to pack their pills.

“For someone like me who travels a lot, or can even spend the night at my workplace … I was always forced to carry the tablets [PrEP] in my bag. But with the injection, once I am injected, it is now just in my body, and so all I have to do is just wait for the next appointment, and then I go back. This has really made my work easy.” (45 y.o. male, Kenya)

The injection improved adherence for those who had a challenge taking pills daily, whether because of an unstable home or work life, or because they feared taking pills in general. “When the provider told me that there would be an injection, I was happy… there were times when I would travel and forget to carry my pills. I felt that they had helped because once you get the injection you are sorted whether you travel or at home.” (39 y.o. female, Kenya)

Participants felt that it was easier to remember the injection appointments than it was to remember to take pills daily at the correct time. They also liked the shorter time lag between the injection and onset of protection compared to PrEP, while also experiencing fewer side effects: “I accepted, knowing that the injection would work for me quickly because it would easily mix with my blood, unlike the pills that would cause nausea when I eat or the pill becoming bitter when I break it to swallow.” (44 y.o. female, Uganda)

#### 3.1.5 Empowerment

CAB‐LA was also preferred by women who had a challenge negotiating condom use. It was especially beneficial for sex workers, for whom negotiating condoms could mean failing to earn money. For others, the injectable option also alleviated worries about taking pills before engaging in unprotected sex, or concerns that providers might be unavailable and cause a delay in starting PEP:

Men are very stubborn; they always want to sleep with you without any protection, and every time you suggest the use of a condom, men tend to deny you money. And because of the need for money that I have, I can't say no to unprotected sex… I used to feel worried every time I was going to meet a man, I would fear such a man seeing my PrEP pills. So when they introduced an injection, I had to go for it – you know, with the injection, no one can tell that you are on any medication. (30 y.o. female, Uganda)

Participants reported having chosen CAB‐LA because their spouse had multiple partners, had another partner who was believed to be promiscuous or of unknown HIV status, or a partner who refused to test. Women felt they had little control over their partner's behaviour, but were gratified that they could take action to protect themselves: “… the health worker assured me that even if he sleeps around, I should be confident that I am safe and will not get any problems.[…] Now that I have the injection, I do not have to worry about that. I have peace.” (36 y.o. female, Uganda) Women also reported that by using CAB‐LA, they were not worried about their spouse coming back unannounced, if they had stopped taking PrEP, thinking they were not at risk because the spouse would be away for several months.

### 3.2 Benefits

Participants also cited newly discovered benefits of CAB‐LA after having used it. These included opportunities to more regularly test for HIV, feeling empowered to have a wider choice of HIV prevention options, fewer side effects compared to oral PrEP, and its familiarity and compatibility with injectable family planning methods.

#### 3.2.1 More frequent testing

Participants regularly tested for HIV when they came for their injection appointment, thereby confirming their HIV status on a regular basis: “Since I started using injectable PrEP, I feel confident: […] I can know my HIV status every time.” (23 y.o. male, Kenya) Repeated negative HIV test results also reinforced their beliefs in the efficacy of CAB‐LA. As one woman noted, “Given the time I have spent receiving this injection and that every time I get tested I am HIV negative, I strongly believe that this drug works.” (26 y.o. male, Uganda).

#### 3.2.2 Choice

Participants reported liking the simple fact of knowing that different HIV prevention options were available. The 23‐year‐old man mentioned above noted that when he tests negative, “I know at the back of my mind that there are ways of HIV prevention other than condom use.” The opportunity to choose between methods gave participants a sense of freedom to make the health decision that would be appropriate to changing circumstances in their lives, in contrast to previous experiences of providers making decisions for them: “…based on how he [the provider] told us about the injection, I felt it was the best method for me to use. I felt good because he left the decision‐making part to us…” (48 y.o. female, Uganda)

#### 3.2.3 Fewer side effects

Some participants also liked the fewer side effects they experienced with CAB‐LA, unlike the oral pills, which participants reported caused nausea. Side effects they did report were different (and more tolerable), most of which resolved after consulting with the provider or after the first injection, as we report below. Participants felt this made CAB‐LA easier to use compared to oral PrEP.

#### 3.2.4 Compatibility with other reproductive health options

Several participants compared CAB‐LA to family planning methods like the quarterly Depo‐Provera shot. “The same way I am adhering to the family planning method, I do to it [CAB‐LA].” (32 y.o. female, Kenya). Another reported, “I am so happy about it. I now want them to bring one that can be injected after every five years… like the one for family planning…Please make sure you inform the people concerned that we need an injection that can last for five years.” (23 y.o. female, Kenya).

### 3.3 Drawbacks

Despite the stated benefits, participants also mentioned features of CAB‐LA that they disliked. These included temporary pain at the injection site, fear of needles and desire for even longer‐acting injectable PrEP. Some participants found the clinic schedule did not align with their work schedules. Others expressed fear that CAB‐LA would be unavailable after the study ended. Among the 10 participants who discontinued CAB‐LA, reasons included travel out of the area, increased physical activity due to farming seasons, blood draws, a potential allergic reaction (rashes) and pregnancy (Table S2).

#### 3.3.1 Temporary side effects

Many participants complained of pain and swelling at the injection site, although this was short‐lived and could be kept secret. Pain was often experienced after the initial injection and reduced with subsequent injections. Providers gave participants painkillers or advised warm compresses:

I cannot walk for two days after getting the injection. … <the providers> said I should boil hot water and use it to compress it. … I did not use the warm compress the first time, and I was in pain for 2 weeks; when I use the warm compress, as the provider mentioned, it takes 5 days, and then I am okay. (21 y.o. female, Kenya)

Despite these side effects, participants generally did not consider them cause enough to stop taking CAB‐LA. Some exceptions occurred, as with a participant who stopped taking CAB‐LA:

“The injection is so painful such that I cannot agree to be injected when I am busy with my farm activities since I am not HIV positive [*laughing*]… If someone misses planting even for a week, then they may lag behind during that season, and they may not have as good harvest as others due to poor timing.” (27 y.o. male, Kenya)

A few participants experienced exhaustion, drowsiness, dizziness, and, in one case, fever, which they attributed to CAB‐LA, all of which participants reported could be addressed sometimes simply by eating well:

I realized that injectable PrEP wants someone to be eating a lot of food [*laughing*]. When I started it, I could feel exhausted and dizzy because by then I was not in a position to get enough food. However, when I started getting enough food, I never felt anything like feeling exhausted or dizzy. That is when I realized that it requires someone to be eating well. … It happened during the farming season when we were busy with our farm activities. (23 y.o. female, Kenya)

#### 3.3.2 Needles and blood draws

Some participants had challenges with CAB‐LA due to fear of injection pain. While some participants learned to lie down or look away to avoid seeing the injection process, others found their fears dissipated after provider counselling and the first injection: “I was scared when they first told me about the injection … but the provider counselled me and told me that the injection was not painful. I also felt that the injection was not painful when I got it and that made me happy.” (30 y.o. female, Kenya).

Some participants also felt using CAB‐LA required more blood samples compared to oral PrEP, as one man who stopped CAB‐LA noted when listing CAB‐LA's drawbacks: “the main one is accessibility, it is only found at the clinic. Secondly, the blood sample collection. … [With oral PrEP], they only collected one bottle, but with injectable PrEP, they collected three…[*laughter*].” (27 y.o. male, Kenya). While he found blood draws unpleasant, his underlying reason for stopping concerned unpredictable mobility (described below). Similarly, fear of needles did not stop participants from coming for subsequent appointments.

#### 3.3.3 Duration

Some participants said they would prefer a method that would last longer than 2 months: “I like it so much and I would suggest, if possible, let them bring an injection that can last for five years. Once I have received the injection, I am just relaxed.” (30 y.o. female, ANC cohort, Kenya)

#### 3.3.4 Concerns about access

During this study, CAB‐LA was only offered at study clinics, posing a challenge to mobile participants or those who incurred transportation costs to the clinic. Participants also switched back to oral PrEP if they were away from the community for more than 2 months due to work and were unable to return to the home clinic:

I told [my provider] to switch me to oral PrEP because I am ever mobile […] CAB‐LA was good because I may forget to take my pills – oral PrEP – but with injectable PrEP, I am safe once I have received the injection. The only disadvantage of injectable PrEP is that it is only received at the clinic. I stopped injectable PrEP or switched to oral PrEP because of that reason: I am not always at home. The cost of transport is also high to get me from where I may be to this clinic, so I still prefer oral PrEP despite the injectable PrEP being the best prevention method. (27 y.o. male, Kenya).

Participants also noted unpredictable travel might cause them to miss a previously scheduled injection appointment. In these cases, oral PrEP was advantageous because it could be carried on the road during longer journeys.

Finally, like the participant cited in the epigraph at the start of this article, participants were understandably worried that they would not be able to access CAB‐LA after the study came to an end: “The provider just asked if I was willing to use the injection, and I also asked them, ‘Will you tell me that the injection has come to an end as well one day? If it will end let me continue taking the pills, but if it will be forever, then let me use it’. That is all I asked them.” (34 y.o. female, Kenya).

## 4 DISCUSSION

This descriptive qualitative study explored clients’ experiences with CAB‐LA within the context of the SEARCH HIV Dynamic Choice HIV Prevention studies in Kenya and Uganda, aiming to identify clients’ reasons for choosing CAB‐LA, experiences with intervention delivery and perceived impact of CAB‐LA in their lives. Our findings illustrate how CAB‐LA uptake overcame many known barriers to oral HIV prevention uptake and persistence. Clients were excited by CAB‐LA's novelty and strongly believed in its efficacy; client trust in providers and in the “proof” of efficacy they felt with repeated HIV‐negative test results while using it facilitated such beliefs. Participants reported two key advantages of CAB‐LA over PrEP: first, CAB‐LA could be used clandestinely, relieving those who did not feel safe disclosing HIV prevention method use and those whose partners or other family members had actively dissuaded or forbidden them from using PrEP. CAB‐LA also offered improved protection from HIV stigma, being visibly distinguishable from ART. Second, these participants felt a heightened sense of safety and protection from HIV acquisition with CAB‐LA because of its easier adherence schedule. Appointments for injections were rare enough (monthly, then bimonthly) that they could be kept, especially with appointment reminders.

Our “real‐world” findings confirm results from the HPTN 083 and HPTN 084 trials among men who have sex with men (MSM) and transgender women who have sex with men (TGW) in the United States and Brazil [12], and cisgender, heterosexual women in Malawi, South Africa, Uganda and Zimbabwe [10]. Like women from Southern Africa, our participants shared similar feelings about the welcomed confidentiality of injectable options. Similar to MSM/TGW in HPTN 083, the challenges of transportation, irregular schedules and pain (at injection site) requiring reduced physical activity were also mentioned by men in our study and indeed led some men to discontinue use of CAB‐LA. Pain and swelling at the injection site, for both men and women, was also reported in these other studies.

Prior research by our team [21, 22] and others [23, 24, 25, 26] has documented both enthusiasm for oral daily PrEP and challenges to persistence, like PrEP‐associated HIV stigma and partner prohibition of PrEP use. Daily pill burden, size, smell, side effects and challenging one's identity as a “healthy person” also present barriers. Moreover, with HIV seen as less severe compared to other diseases, HIV prevention benefits can be outweighed by perceived costs. For individuals facing these barriers, CAB‐LA is a valuable alternative prevention option.

This study specifically focused on the experiences of participants who chose CAB‐LA; we did not interview those offered CAB‐LA but who preferred continuing oral PrEP or PEP. Thus, a limitation is that we lack specific information on why clients preferred other HIV prevention methods over CAB‐LA. However, we did collect data from individuals who discontinued CAB‐LA and switched to other methods. The reasons for this discontinuation ranged from workloads and transportation challenges to responsibilities outside the catchment area. Additional reasons included pregnancy (a concern also in the HPTN 084 study) and one case of an allergic reaction [10]. Injection site pain and swelling as a reason for discontinuation appeared more closely linked to how it compromised one's ability to do physical labour in gardens and on farms than to lingering pain; most participants reported the pain decreased with subsequent injections or subsided after a few days.

The study built on the SEARCH Collaboration's prior work to establish a patient‐centred care model wherein providers were trained to provide care in a welcoming and friendly environment, and to help patients overcome uptake and adherence barriers [27, 28, 29]. Thus, these findings report on experiences with prevention products that are not affected by a poor quality of care delivery (which, when operant, may present additional obstacles). Findings from these participants underscore that method choice is paramount for all clients: as we have previously documented [30], client needs and preferences may vary by seasonal work demands, changing relationship circumstances, perceptions of risk and travel schedules. For some mobile individuals, CAB‐LA conferred benefits over oral daily PrEP that had been hard to manage during travel, while for others, an extended prescription for oral PrEP was a more flexible option. No one method “solves” all challenges to engagement in HIV prevention; rather, the addition of CAB‐LA to the prevention toolkit expands the options for individuals to find what works for them.

An important population that stands to benefit from continued access to CAB‐LA are women whose partners, spouses or in‐laws forbid bringing HIV prevention medications into the house or who threaten violence if they discovered these women using it. The multiple instances women reported of such incidents confirm prior research from the region showing that partner non‐support is a major barrier to PrEP uptake and persistence for women [22, 25, 31, 32, 33]. More often, in a context where multiple partnerships are common and households are geographically stretched, women reported not being able to anticipate potential risk periods because they did not know when a mobile partner would return home, and thus had difficulty planning their oral PrEP regimen. For these women, CAB‐LA conferred long‐lasting protection that gave them a feeling of assurance. The most extreme cases of a lack of control occur in cases of rape. Like with PEP, women highly valued CAB‐LA over PrEP as a means of preventing potential exposure to HIV via sexual assault [34]. Finally, findings also supported men's strong motivations to use CAB‐LA. While men did not report a fear of violence from female partners, many did fear disapproval, blaming, mistrust and conflict if their partner discovered them using an HIV prevention method. Like women, men highly valued the privacy of CAB‐LA and the option for when and to whom to disclose its use.

Importantly, our study shed light on the continued need to educate clients and communities about HIV prevention methods; health literacy, especially related to prevention products, will continue to facilitate the success of HIV prevention efforts. All participants voiced a desire for more health education in their communities to promote knowledge and acceptance of the full range of HIV prevention methods.

CAB‐LA holding promise for expanding uptake of HIV prevention and enhancing protection through increased ease with adherence is supported by our findings. However, as recently noted [32, 33], CAB‐LA, Lenacapavir and other products in the prevention pipeline are stymied by widespread access and implementation‐related barriers, due to pricing, policy changes and major funding reductions in the US administration. A potential breakthrough in ending new HIV acquisitions remains at hand, but only if commitments are made to make these novel HIV prevention medications accessible and available to those in need.

## 5 CONCLUSIONS

Several known barriers to HIV prevention uptake and persistence were overcome with CAB‐LA. In the context of continued HIV‐related stigma, CAB‐LA met some participants’ preferences for a product that permitted prevention to be visibly distinguishable from treatment, enabling prevention uptake to feel safer; the privacy of injections affords this desire. Moreover, adherence was more easily achieved with CAB‐LA compared to PrEP, boosting confidence in prevention efficacy.

## COMPETING INTERESTS

All authors declare no competing interests, financial or otherwise, related to the work submitted for publication. DVH received non‐financial support from ViiV (drug donation for the study).

## AUTHOR CONTRIBUTIONS

Conceptualization: CSC, AO and JJ‐P; Writing: AO, JJ‐P, CSC and JK; Data collection: LO, AO, TOA, CA and FA; Analysis: AO, JJ‐P, LO, CA, TOA, FA, AM, AB and CSC; Supervision: JK, JA, CSC, HS and NS; Qualitative study administration: JJ‐P; Funding acquisition: DVH, MRK and MP. LBB, MC, and GC and all other authors contributed to review and editing.

The team‐based analytical approach was enriched through a lens of local experience by qualitative study research team authors who had a first‐hand understanding of the study setting (AO, CA, FA, LO, TOA). The research team members who gathered data in the local languages Dholuo (LO, AO, TOA) and Runyankole (CA, FA), also engaged in data analysis, and confirmed interpretations of the data to which additional team members contributed (JJP, CSC). This ensured that the translation and interpretation of the data respectfully captured the nuances of participants’ voices. The researchers acknowledge the possibility of contested realities and the relativity of this analysis.

## FUNDING

Research reported in this publication was supported by the National Institute of Allergy and Infectious Diseases of the National Institutes of Health, under Award Number U01AI150510 (Havlir/Kamya/Petersen). ViiV provided CAB‐LA for the study.

## DISCLAIMER

The content is solely the responsibility of the authors and does not necessarily represent the official views of the NIH.

## Supporting information



Supporting Information File 1: CAB‐LA IDI Guide Supplementary File
Word document of the questions contained in the CAB‐LA interview guide that the team of trained qualitative interviewers administered to CAB‐LA study participants who consented to the qualitative interviews.

Table S2. Reasons for discontinuing CAB‐LA

## Data Availability

De‐identified qualitative study transcript data and a codebook will be made available in a secure repository following SEARCH Scientific Review Committee approval of a concept sheet summarizing the analyses to be done, with a signed data access agreement. Further inquiries can be directed to the SEARCH Scientific Review Committee via Carol.Camlin@ucsf.edu.
