# Supplementary material for: Early experiences with usage of long‐acting injectable cabotegravir among adults in rural Ugandan and Kenyan communities: qualitative research from the SEARCH “Dynamic Choice HIV Prevention” intervention trials
Source: J Int AIDS Soc. 2025 Nov 24;28(11):e70059. doi: 10.1002/jia2.70059 (PMC12644244; doi:10.1002/jia2.70059)
Supplement: Supplementary file 1 — Supporting Information File 1: CAB‐LA IDI Guide Supplementary File Word document of the questions contained in the CAB‐LA interview guide that the team of trained qualitative interviewers administered to CAB‐LA study participants who consented to the qualitative interviews. [file JIA2-28-e70059-s001.docx]

**SEARCH-SAPPHIRE: A Multisectoral Strategy to Address Persistent drivers of the HIV epidemic in East Africa**

**CAB-LA Data Collection Instruments**

The objective of social science research in Phase A has been to broadly ascertain the perceptions of DCP interventions among members of target populations and the DCT intervention in mobile populations, the hypertension treatment study, and among health care providers delivering DCP, hypertension treatment, and the Mobility DCT model, vis-à-vis: 1) Experiences with and acceptability of individual elements of DCP/DCT; 2) Key barriers to uptake (clients) / delivery (providers); and 3) Preferences for modifications to improve DCP/ DCT models. The Phase A study was designed to provide rapid insights into key implementation factors that can inform modification of interventions for scale up in Phase B.

This document adds a supplementary instrument to those presented in the Phase A research plan, to account for the roll-out of long-acting (LA) injectable PrEP.

**Phase A continuation instruments in this document:**

Dynamic Choice Prevention trial populations: Long-acting Injectable PrEP Option

1. In-Depth Interview (IDI) guides: Men and Women who have opted for long-acting, injectable PrEP, with specific embedded questions for adolescent/ young adult (< age 21) and reproductive age women, in ANC, OPD, VHT and youth multi-sector interventions.

**[DCP] In-Depth Interview (IDI) Guide 1: Men and women who selected injectable PrEP**

**Cross-cutting and embedded questions for: adolescent/ young adult women (< age 21), antenatal clients, and postnatal clients. Interviewer: adapt language for type of client as indicated. After completing informed consent procedures, the interviewer uses the following guide to interview study participants. Interviewer will draw upon the suggested questions and prompts in this guide, but may insert additional follow up questions and probes as needed to explore topics fully. Instructions to interviewers are italicized. Note to interviewer: Ensure privacy and comfort of participant, and enable audio recorder after informed consent and before starting interview. Do not read topic headers aloud. Reiterate consent and confidentiality protections as needed during the interview.*

The purpose of this interview is to find out your opinions about and experiences with accessing health care and prevention services, and specifically your experiences with services provided by health care providers working with the SEARCH-SAPPHIRE study. The information that you share with us will help us to improve services in order to help other [women / men] like you. I want to remind you that as we discussed during the informed consent process, the information you share will be kept strictly confidential and will have no effect on the services you receive, so thank you in advance for your open and honest responses.

Today I will ask you questions about your experiences with being offered health services, about the choices you made about which services you wanted, and about the interactions you had with health care providers. I’ll also ask you about issues in your life and in the community that either help or hinder you from accessing services. Finally, I’ll ask your opinions about how these services could be improved. Do you have any questions for me before we start? [*Address questions, start audio recording and begin*.]

**Preliminary Questions**

*Household & family context*

1. Let me start by asking you about your current living situation. Where do you stay currently?
2. Who are you living with? [probe to establish household structure and composition]
3. And do you have another place that you consider to be your home? Tell me about that.

*Livelihoods & mobility*

1. How long have you been living where you stay now?
2. Do you have any plans to move/change residence any time in the next few months or year?
3. Do you ever spend time away from your main place of residence, for purposes of work, or schooling? Tell me about how you tend to divide up your time. [probe for proportion of time participant typically spends in home community vs. away from home; anchor to past three months if needed]

*Social support*

1. Now I’d like to ask you about the people in your life who you turn to for help and support. Who helps you make ends meet, in terms of helping you pay for food or any other basic needs? [Probes: Probe for livelihoods and/or parental, spouse or family support.Tell me about that person. Is there anyone else?]
2. Tell me, is there anyone in your life who you feel you can confide in? [Probes: Tell me about that person. Is there anyone else?]

**I. Experiences with DCP counseling, method/service preferences and decision-making to go on INJECTABLE PrEP.**

1. I want to continue by asking you some questions about the services that you have been accessing [in this program]. Can you confirm for me, what are the main reasons you have been engaging with services offered by this program? [Probe for type of services]. What other types of services have you been receiving?
2. Now, tell me about your most recent visit when you were counseled about various options for services you could receive and learned about long-acting, injectable PrEP. What kinds of health services and options did the provider tell you about and how was injectable PrEP explained to you? [*probe expanded PrEP, PEP, condoms, HIV testing, and long-acting, injectable PrEP options*].

- Had you heard about long actng PrEP before, from another source other than the provider? Tell me about that.

1. How did it feel to be offered this new option?
2. Tell me any questions you remember having about injectable PrEP at the time. How did you ask the provider about that? What else did you wonder about after the provider answered those questions?What are some things you still wonder about? Tell me more about that.
3. Tell me please about the conversation you had with the provider, when you were trying to decide whether injectable PrEP was right for you. What kinds of questions did the provider ask you? Was it easy, or hard, to talk about whether any of these services/options/methods would be right for you?
   - What were you told about injectable (long-acting) PrEP?
     1. What did you think about it?
     2. How did you compare it to oral PrEP?
     3. How did you compare it to PEP?
     4. How did you compare it to condoms?
     5. What did you think about other options to prevent HIV or options for sexual and reproductive health care compared to injectable PrEP?
   - What were you told about how often you would need to come to the clinic to receive injections for injectable PrEP? How has that been working for you?
4. Now I want to ask you about how you felt treated by your health care provider. How respected did you feel? How worried did you feel about being judged? Tell me about that.
5. What did you like about the way that your health care provider counseled you? What was difficult or uncomfortable about the discussion? Tell me about that.
6. Could you please tell me about where you have received services as part of this study? [e.g. clinic, household, other community location?] What options were you given as to where to have follow-up visits? If you’ve changed locations, what led you to change locations?

- Where do you go to get the injections for injectable PrEP? How do you feel about receiving it at this location? How was that experience? What do you like about it?

**II. Method/services satisfaction, barriers and facilitators to uptake and maintenance, and social networks**

1. Tell me aboutwhen you decided to switch to injectable (long-acting) PrEP?
2. What are the main reasons you decided to take up injectable PrEP? What other reasons? What do you like about it?? What are the drawbacks of this method? What in your mind are the main benefits of it?
3. When you were deciding to take up injectable PrEP, how confident did you feel about your ability to continue to use it over time?

- How confident were you that it would be effective?
  - What concerns do you have about whether or not it will be effective, if any? [i*nsert as needed: to keep you protected from HIV infection*]
- *How easy or difficult was it to begin injectablePrEP?*
- *What challenges did you experience around injectable PrEP?*

1. Now, tell me about any other service the provider offered to you, that you thought you might be interested in, and considered it, but decided against it. [*Probe for methods/options*]. What were your main reasons for not opting to take up that [*method/option*]? What else?
   - What other locations besides the clinic would you suggest to someone who wanted to offer a long-acting injectable prevention method? Why those locations?
2. Now I want to ask you about how your choices of services changed over time in the past few months. First, tell me, how did you feel when your provider told you from the beginning that there were choices for prevention, and you could adjust your choice as needed?

- [*Probe as needed*]: How helpful was it to know this? What questions did you have at the time? Did you anticipate that new methods would be added to your range of choices?

1. What method(s) have you changed since you first started? Were there other methods or services that changed? What about locations where you receive services?

- [*If changes in choices of services/methods: probe by method/service changed*. *If locations changed, rephrase accordingly*]:What led you to change methods?
- What information from your provider helped you to make that change? Was there other information you would have liked from your provider to help make that decision?
- [if they have continued using CAB-LA]: How likely is it that you’ll stay with injectable PrEP? Why (or why not)?
- How satisfied are you with injectable PrEP?
- [if they they discontinued]: How likely is it that you would try again to use injectable PrEP?

1. Now I’d like to ask you more about your experiences with injectable PrEP. Tell me about any problems like side effects or physical discomforts you’ve had while using it.

- [*If there were problems*:] What help were you able to get from a health care provider about this? How did that go?
- How did this lead your stopping the use of injectable PrEP, or how did it lead to you still using it? Tell me more about that.

1. As a result of enrolling in this program, tell me about any discussions you have had with your friends about HIV prevention options.

- Have any of them decided to start HIV prevention? Tell me more about that.
- How likely are you to recommend injectable PrEP to a friend? Why (or why not)?

1. What concerns did you have, if any, about other people in your life knowing that you were using injectable PrEP? How have you managed that?
2. How familiar are people in your community with injectable PrEP? What do they think (or what might they think) about it? Who thinks this way? Why?
3. Who are the people in your life who you trust to talk about your use ofinjectable PrEP? [Probe whether includes intimate partner(s)]. Why are you able to talk with that person/those people? Which people in your life might you feel you have to keep this a secret from? Tell me about that.
4. What was the biggest challenge you faced in staying on some form of HIV prevention? What more could be done to make it easier for someone like you to use HIV prevention methods?

**III. Recommendations to improve DCP model**

1. We are getting to the end of this interview, and I now want to ask for your recommendations about how the services you’ve been receiving could be improved. First, what are the main things that could make it easier for you to access HIV prevention services, especially injectable PrEP, if anything?
   - [*Probe for more service delivery modalities*] What other ways of delivering prevention services would you suggest?
   - What about mobile clinics?
   - What about sites set up near trading centres?
   - What about having clinic registration cards that can be used at more than one clinic?
2. Why would you want to continue to receive care where you are accessing care now, or why would you prefer to go elsewhere?
3. What can your current provider do to make it easier to talk with her/him about the services that you need? Tell me more about that.
4. What other health care services do you wish it were easier for you to access? Tell me about that.
5. Now please share anything else with me that you would like our study team to know.

*THANK STUDY PARTICIPANT FOR THEIR TIME AND END INTERVIEW.*
