# Supplementary material for: Early experiences with usage of long‐acting injectable cabotegravir among adults in rural Ugandan and Kenyan communities: qualitative research from the SEARCH “Dynamic Choice HIV Prevention” intervention trials
Source: J Int AIDS Soc. 2025 Nov 24;28(11):e70059. doi: 10.1002/jia2.70059 (PMC12644244; doi:10.1002/jia2.70059)
Supplement: Supplementary file 2 — Table S2. Reasons for discontinuing CAB‐LA [file JIA2-28-e70059-s002.docx]

**Supplementary Table 2. Reasons for discontinuing CAB-LA**

| **Reason** | **Sex** | **Age** | **Country** |
| --- | --- | --- | --- |
| "We normally just get medication; that is all I can say. I tried using the injection and the swelling lasted for a month and I decided to stop for some time. I continued taking pills and then I became ill and stopped using the pills as well and took about a month without using the pills; it now about one and a half months since I stopped taking medication. I think CAB-LA is okay but the swelling is the problem." | F | 23 | Kenya |
| "I told them that I have decided to take a leave from using the injectable PrEP but I will just make a come back later. ...I was being injected like immediately, it was a short duration between one injection and the other. There was another concern about the amount of blood being withdrawn; they are taking a lot of blood and this has concerned me a lot to an extent that in case I feel something like headache then I will just think of my blood that was withdrawn. " | F | 37 | Kenya |
| "What made me miss, the first injection that I took, I later fell sick and it was a serious sickness, at first after taking it, I got swollen and the one who had injected me was M… and told him about it and he brought me some medicine to smear on the affected part to un swell, and after un swelling, I developed other things on the body, I developed rashes and some funny stuff on my body, I would scratch my body, the feet even under it all developed rashes, and people in my community who would see me would tell me that it’s a traditional illness associated with having twins. I kept quiet and also went into deep thoughts. I wondered, whether it was because of the injection? What was it?" | F | 37 | Uganda |
| "I will stop it, if I get a person <partner> that I trust." | F | 24 | Uganda |
| "You know our situation in the villages. Sometimes there is no time, there is no transport. So at some point the health worker recommended that I switch back to the pills because I could take them for a while." Later "Looking at everything that she told me, I saw that the injectable would be an easy option for me. But after two months, things took a toll on me. I got so busy and I did not have the time to come here. So when I came here, I talked to the health worker and told her that I would not be able to continue with the injection because I do not have the time to come to the health facility. She said that was no problem, I could still go back to taking the pills." | F | 32 | Uganda |
| "I stopped CAB-LA because by that time, my father was sick and I was attending to him. I could not leave him alone at the hospital to come to the facility for my appointment. This made me to miss my appointment. When I came back, we reviewed it with J (study provider), and I told him to switch me to oral PrEP because I am ever mobile; maybe the next day I will not be around yet it was supposed to be my injection day. Meaning I will continue missing my appointments and he cannot cater for my transport to and from the clinic when I come. Again, I may not find time from work to come to the clinic. The other reason he could not refer me to any hospital or clinic because CAB-LA was just being offered within Homa-Bay and Migori Counties at designated clinics; it was not available in all the clinics. " | M | 27 | Kenya |
| "I was told that injectable PrEP is part of or similar to oral PrEP. Then I was offered the injection that it is also good and I can stop oral PrEP and use the injection and I will still be protected. I only got one injection. ...I was so busy in school and this is why I missed my scheduled appointment. During that time, we were supervising the examination and being a private school, we were ever busy." | M | 30 | Kenya |
| "I like working in my farm and one day when I went to my farm as usual, I became so tired and I asked myself, “What is the problem with me?” and since I shared about it with my wife, she advised me to stop the injection and observe how things will work out. Thereafter, I stopped the injection and when I went back to the farm again, I was just okay. I worked well then, I thought this injection was the problem. I only received the injection once and I did not go for another refill." | M | 59 | Kenya |
| "The injection is so painful such that I cannot agree to be injected when I am busy with my farm activities since I am not HIV positive…laughter. No, I cannot accept it during my busy time. ...Laughing…since farming activity can go for at least three months; it means I missed it for that whole season. In case someone receives the injection at that time, again s/he may miss to complete his/her farm activities for that entire season." | M | 27 | Kenya |
| "After having those transport challenges…at the time I had brought my wife to the HIV clinic, she had come to ask for a transfer to the health facility near our home, so I went to K. to explain to him my situation. So K. said that he would inform his bosses about my situation and he would let me know. I missed my next visit for the third injection. So K. made effort to talk to his bosses and they agreed and gave him transport to always find me in the community and he always comes and we meet at [a Health Centre III]." | M | 26 | Uganda |
